# Service innovation practices and customer loyalty in the telecommunication industry

**DOI:** 10.1371/journal.pone.0282588

**Published:** 2023-03-10

**Authors:** Ernest Awuku, Paul Mensah Agyei, Eric Gonu

**Affiliations:** 1 Department of Business Studies, College of Distance Education, University of Cape Coast, Cape Coast, Ghana; 2 Department of Marketing and Supply Chain Management, University of Cape Coast, Cape Coast, Ghana; Al Akhawayn University in Ifrane, MOROCCO

## Abstract

The study sought to assess differences in innovation practices in the telecommunication industry, customer perception of service innovations, and how service innovation practices influence the loyalty of mobile subscribers. A quantitative research approach was adopted to study 250 samples from active subscribers of the leading mobile telecommunication companies in Ghana. Descriptive and regression analytical approaches were used to analyze the study’s objectives. The result indicates service innovation practices significantly influence loyalty. Innovative service concepts, innovative service processes, and new technologies significantly influence customer loyalty with the latter having the strongest influence. The study contributes to the scanty literature on the mentioned subject within the Ghanaian context. Additionally, this study focused on the service sector. Despite the sector’s contribution to the world’s Gross Domestic Product (GDP), previous studies have largely focused on the manufacturing sector. Based on the findings, the study recommends that the management of MTN, Vodafone, and Airtel-Tigo in collaboration with R&D and Marketing departments must invest financial and cognitive resources to develop innovative technologies, processes, and services to address the service convenience, efficiency, and effectiveness needs of customers. The study further recommends that financial and cognitive investment should be based on market and consumer research, and customer interaction. This study recommends similar studies using qualitative research methods in other industries such as banking and insurance.

## Introduction

Service innovation is one of the strategic approaches for firm survival and has gained lots of attention not only from academic scholars but also from industry experts [1, 2]. However, the literature on service innovation is dearth due to the erroneous perceptions in the literature and among practice that service innovation is a preserve activity of manufacturing entities. Again, the mobile telecommunications networks in Ghana are hit with frequent service challenges and customers continue to raise concerns about the service delivery by these companies [3]. [4] blamed this on a lack of knowledge of how service companies can use their service innovations to drive customer satisfaction and loyalty. This study seeks to provide knowledge on service innovation in the service industry by examining the effect of service innovations on the loyalty of mobile subscribers in Ghana.

Innovation is generally described as a catalyst for businesses to keep pace with market changes and drive customer satisfaction, loyalty and performance [5]. The literature explains that in an agile global marketplace where consumers seek better value, innovation is essential for customer satisfaction and loyalty [6]. Scholars in the area of innovation have further mentioned that businesses can use innovations strategically to compete in local and global markets [7]. [7] explained that innovation allows firms to refocus and adapt their orientations to the changing market trends and dynamics and also provides superior customer value which leads to higher firm performance. Even far back from the time of Schumpeterian [8], innovation is acknowledged as a great contributor to value creation, giving businesses a competitive advantage.

One important strand of innovation in service innovation. Service innovation involves change or renewal in the service offering, service delivery procedures and processes that are either new to the company and/or to its customers [9]. It also means improving, expanding or adding new features and service portfolios or and/or improving existing services and processes [5]. In a competitive service environment, service innovation is one of the strategic routes for companies to survive and enhance their growth. This is because the practise of service innovation introduces new services and processes that cater to shifting preferences, tastes, and choices.

Scholars have, therefore, explained that service innovation support businesses to develop new or improve services as well as the delivery processes e.g., [10]. Innovative service organisations can launch new and improved services efficiently, at a lower cost and meets the needs of customers. For many years, some scholars [11] have pointed out that the ultimate goal of service organisations has been to get new customers and also to maintain existing ones. The key to customer loyalty is the ability of the firm to provide products tailored to meet the needs of its customers [9]. This is to say that service innovation creates customer loyalty, hence low marketing costs [11], and favourable word-of-mouth recommendations [12].

In Sub-Saharan African countries including Ghana, intense competition in the telecommunication industry have pushed telecommunication firms to build robust innovative service offering [13]. Recently, Airtel Ghana and Tigo Ghana merged to boost their service delivery and innovative capacity. In Ghana, the companies in telecommunication industries including MTN, Vodafone, Airtel-Tigo and Glo continue to introduce innovations to increase customer satisfaction. Innovations such as; international roaming services, internet services, mobile money services, alert services, mobile banking services, utility payment services, teleconferencing facilities, and many others [14]. Innovations from telecom operators in Ghana are increasing astronomically. These innovations offer customers a variety of choices but the rate of changes in the industry is rendering existing innovations irrelevant [15]. The high competition means that telecommunication companies must constantly improve their innovative practices to increase customer satisfaction and loyalty to service offerings.

In the Ghanaian telecommunication industry, innovations such as mobile money services and utility payment services have become common. It has therefore become a common phenomenon that the rate of innovation in the telecommunication industry is outcompeted within the shortest period.

The rate of innovation in the telecommunication industry means that telcos must enhance their innovation practices to increase customer loyalty. [16] note that to keep customers loyal to a particular innovation, the items must carry the "promise" that a good or service possesses the right features, qualities and characteristics which make it special or unique to satisfy his/her need. The innovation can help address a problem or even delight the customer before it can drive customer loyalty. Given this, organizations must understand the influence of their innovation practices complemented by effective marketing programs that consumers can appreciate, trust and gain the needed satisfaction that will drive their loyalty to the brand.

This has necessitated the emphasis on innovation as the way forward for Ghanaian telecommunication industry firms to achieve increased customer loyalty. However, about 90 per cent of new products that are introduced into the market fail to achieve business objectives because the innovations do not meet the need of the customers [17]. Constant innovation practices are required for businesses to develop better offerings that stand the test of time and drive customer loyalty. To understand these issues from a Ghanaian perspective, the study uses the telecommunication industry in Ghana to assess innovation practices and how it leads to customer loyalty.

The literature on service innovation is dearth because scholars have perceived that innovation is a preserve and sole activity on manufacturing enterprises. As a result, some studies have recognized that the practice has not gained much attention within service business settings [18], as manufacturing continues to dominate innovation literature [19]. Quite profoundly, a taxonomical review of the extant literature by [19] revealed a paucity of studies on innovation while scholars have barely focused on innovation. There is a call by scholars, for a critical examination and extensive research into service innovation.

It appears that a considerable number of innovation studies are largely focused on the manufacturing sector [19, 20]. The subject of innovation in the services sector, on the other hand, has received relatively limited attention in academic spheres [19, 21]. The few available studies on service have not given the needed attention to the telecommunication industry [22]—‘post office’; [23]–‘banking’; [24]- safety industry.

However, given the tremendous contributions of the services sector to the world’s Gross Domestic Product (GDP) and the economic activities of most countries [7], there is a need to examine the state of innovations in the telecommunication service industry, and its impact on customer loyalty from a scholarly standpoint.

In this fiercely competitive mobile telecommunication market [25], several service innovation practices such as mobile money, and utility payment have been introduced [14]. Nevertheless, mobile telecommunications networks in Ghana continue to experience service deficiencies while subscribers continue to express their displeasure about the services offered by telecommunication companies [3]. [4] blamed this on a lack of knowledge of how service companies can use their service innovations to enhance customer satisfaction leading to customer loyalty. This lacuna is worrying considering the rate of innovation in the telecommunication industry [26]. It has become important for telecommunication firms to understand how they can leverage their service innovation practices to enhance customer loyalty [4]. Extant literature on innovation from Ghana [18, 25], did not focus on customer loyalty.

Less is known about innovation in the service market especially the telecommunication sector due to the erroneous perception that innovation is a preserve of the manufacturing firm. This study has become very important because the service industry is becoming competitive and telecommunication companies cannot afford to experience customer turnover, hence the need for empirical knowledge for decision-making.

This study examined this gap by specifically, assessing the differences in innovation practices among telecommunication companies in Ghana, assessing customers’ perception of service innovative practices in the telecommunication industry and examining the effect of service innovation practices on the loyalty of mobile telecommunication subscribers in Ghana.

The remaining sections of the paper focus on materials and methods, results and discussion, conclusion and recommendation. The paper ends with future research directions.

## Theoretical foundation

The disruptive innovation theory can be traced to [27] who suggested that disruptive innovation is associated with the capability of a person or an entity to destroy and cause changes in an existing value and standards in an industry or environment. [27] further noted that the destruction results in drastic modifications and changes in the industry resulting in the development of innovations. Further to the position by [27], other scholars such as [28] have contributed to the development and popularization of the theory. For example, [28] attributed disruptive innovation to technologies and explained the concept as a source of value differentiation and superior value. Another view was espoused by [29] who said that the changes that emerge from disruption often have an attribute that focuses on a particular niche market.

The telecommunication industry is very competitive, thus forcing operators to constantly improve their existing innovations. In relating the disruptive innovation theory to this study, the researchers argue that service operators must improve on existing services to retain their existing customers. While service providers need to develop new service offers, they also think of looking at ways to improve the existing ones to target a new market. It is a characteristic of the Ghanaian industry where industry operators are constantly improving their existing service innovation. These innovations are served unserved (niche) markets ignored or unexplored.

## Literature review

### Service innovation

The literature on service innovation is yet to achieve a consensus on the conceptualization of the concept of “service innovation” despite assuming significance in organizational performance literature [9, 30]. Scholars and practitioners continue to debate and explore the concept and its application since it is considered a significant driver of socioeconomic growth and development [31]. According to [32] service innovation can be considered from three main domains which include first, “the development of a service or product which is new to the supplier, something which is not available to the firm’s clientele, resulting from add-ons to or modifications in the service concept and encompassing ideas, practices or objects, which are new to the organization and the relevant environment”. This means that the conceptualization of service innovation must include a degree of change or renewal in the service product, processes, and procedures. This change may either be something new known to the customer and/or the company.

Service innovation exhibits some special characteristics which make innovation in the service industry different from innovations in the manufacturing industry [33]. The view by [33] supports the assertion by others to conclude that innovation does not apply to service firms. Other scholars such as [32], noted that innovation in services may be considered from the following dimension. First, within services innovation, the difference between product and innovation is beginning to slim because innovations in service and product usually overlap. For instance, a new service normally goes jointly with a new distribution pattern, client interaction, and quality control mechanism.

Second, service innovations can also be examined from the degree of novelty perspective, which may be a gradual or small change to a large or radical medication. Most importantly, radical innovation results in large developmental innovations which occur in a well-organised system and environment. On the other hand, incremental innovations are mostly small and occur in a less formalised environment. Incremental are mostly of lower novelty and the degree is common they typically entail small modifications in organisational procedures and processes, are easier to develop, and have a lower risk and cost.

Innovation must result in newness in the new concept of services already employed elsewhere, a new market or both [34]. Additionally, a mixture of significant and insignificant modifications or adaption of existing services is also expected [35]. The literature (see [32]) explains that service innovation, being new, novel, incremental or radical must support organizations to develop cheaper, faster, and higher quality services. This makes service innovation a source of business performance [36].

### Service innovation practices of the telecommunication service organization

The Ghanaian mobile telecommunication landscape is principally driven by private companies. Currently, the industry has four operators namely MTN, Airtel-Tigo, Vodafone, and Glo, and recently Surfline whose license only allows the company to strictly provide data services for domestic and industrial purposes on the 4G platform. Until recently, the main services of mobile telecommunication companies were cellular and data services to customers. However, the disruption in the industry caused by intention competition has compelled the service providers to improve and bring newness to their service features, processes and technologies such as mobile voice SMS, mobile money services, audio conference; and directories. They also explore opportunities to develop new service innovations through new offerings, designs and service delivery processes. Indeed, telecommunication companies who have a first-mover advantage with their innovation enjoy.

### Customer loyalty

The concept of customer loyalty has been defined from various perspectives but the central idea is that loyalty is about sustainability and long-term relationship. Customer sustainability and a long-term relationship between a firm and a customer are important aims of every firm that wants to survive and grow in the 21^st^-century telecommunication industry. Customer loyalty, according to [37] also defined customer loyalty as a customer’s level of commitment to continue purchasing a particular service brand regardless of other influences from market competitors to switch behaviour. It is also a sustained satisfaction tied customers’ emotional attachment to a brand that emerges out of the customers’ willingness to maintain the relationship [38].

[39] explained that customer loyalty represents a customer’s attitude to purchase and repurchase services or products, recommend to others and resist other competition brands. When these attitudinal elements are not properly controlled or checked, there is a tendency for the organization to lose such customers to other brands that offer better alternatives. The justification is that organizations that can make their customers loyal derive several attitudinal and behavioural benefits. Loyal customers provide strong positive word-of-mouth and references to other potential customers. These loyal customers create positive business referrals and even serve on advisory committees of the firms. Loyal customers also represent a “fantastic marketing force” which has been considered the best available advertising tool for companies. In this research, the constructs for customer loyalty include recommendations and referrals.

The phenomenon where companies are constantly innovating, coupled with technological development has made mobile phone operators deliver new kinds of services to conventional voice services. Other service concepts, processes and technologies offered by some telecommunication companies in include post and pre-paid services in internet and call services. For example, MTN offers innovation in mobile services which allow mobile users to track their vehicles, do audio calls, roam and mobile money services. The companies have also enabled technologies that allow mobile users to connect instantly through video conferencing, multimedia messaging services, short messaging system (SMS), location-based service TV and radio broadcast and data transmission and internet connection services (GPRS), and video conferencing through fast internet Mobile Broadband.

### Service innovation practices and customer loyalty

The literature from the Global System for Mobile (GSM) Communication Association identified a list of indicators that measures the loyalty of mobile money subscribers. These indicators included network access, service access and service integrity [40].

Empirical evidence by [23], investigated the influence of service innovations on how service delivery influences the loyalty of customers in the banking industry of Ghana. The study found that innovations in the service activities of commercial banks significantly influence the loyalty of customers in commercial banks in Ghana. In the hospitality industry, [41] concluded that differentiation, among brand innovativeness, predicts better loyalty.

In [9], it was established that B2B services-focused firms are less sophisticated in their innovation practices compared to B2B products-focused firms. Again, B2B service firms focused-on innovations that are less explicit. They have fewer innovation expectations and prefer more incremental innovation, spending less time taking their innovation to market. The study further affirmed that the majority of B2B firms practice a hybridized service-product offering to customers meaning that most B2B firms provide both service and product orientation innovations to their customers. This finding is not different from what was identified in the safety industry of the business market. [24] result indicates that service innovation in the form of technology-oriented and co-creation-oriented innovation influence business customer loyalty and performance.

[42] investigated how innovations and customer experiences affect organizational reputation and loyalty of customers. Evidence from the findings suggests that innovation reflects organizations’ positive image. The study also found a significant link between the reputation of the firm and customer loyalty. Similarly, [43] investigated the effect of service innovation on customer retention in the Abokobi-Madina locality and revealed that innovation practices such as mobile money service by mobile telecommunication firms had a significant positive effect on customer loyalty.

## Methodology

### Research design

Based on the deductive approach and objectives of the study, this study adopted the survey research design to examine the relationship between the influence of service innovation practices on the loyalty of mobile subscribers in Ghana. The researchers tend to generalize the findings to the entire population which makes the design appropriate [44]. Survey design is consistent with research purposes that seek to use a large sample from a given population in an efficient approach [44]. A survey design was employed to provide an efficient, precise causal perspective on the issues to generalise the findings.

This study employed an explanatory research approach [45]. This was on the basis that [12] noted that an explanatory design allows researchers to perform a causal assessment of two distinct variables such as dependent and independent variables. The explanation approach was considered appropriate because it best fits the aim of this study which sought to investigate the influence of some service innovation practices and their influence on customers. The literature is vast on service innovations. Measures of these constructs have been vastly explained so this study only seeks to employ these variables and apply them to a different context. Another justification for choosing explanatory is that it provides a comprehensive causal assessment of a phenomenon [12].

### Population

The target population includes active subscribers of the leading mobile telecommunication companies in Ghana in terms of active customer base. The inclusion criteria for the population is that a respondent must have registered on at least one recognized telecommunication company in Ghana. A potential respondent must have an active mobile service number with either MTN, AIRTEL-TIGO, or VODAFONE telecommunication service providers. One main justification for choosing these three companies is that, according to the National Communication Authority’s report, these three companies have the largest number of mobile subscribers based in Ghana. First, MTN has the largest subscriber base of 14,207,778 (MTN). MTN is followed by Vodafone 7,159,556 and Airtel-Tigo 7,264,078 (NCA, 2015). Consequently, these three companies have a total of 28,631,412 mobile subscribers in Ghana and the researchers consider this number as the population.

### Sampling procedure

There is no readily available sample frame of active mobile subscribers in the Cape Coast Metropolis, Ghana. Thus, a non-probability convenient sampling technique was employed by the researchers to target and select active mobile subscribers. These subscribers include subscribers of the top three leading mobile telecommunication in Ghana who has been using their network for the past twenty-four months and is resident of Cape Coast Metropolis.

One reason for convenience sampling is that it will allow targeting of subscribers who are willing and available to participate in the study, and also easy to access in terms of geographical proximity of the respondents [46]. Two years of a subscription is used in the sampling procedure because it allows subscribers to have an opportunity to experience any innovations by their service provider. In terms of sample size, a sample of two hundred and fifty active mobile subscribers will be targeted during the data collection stage. The justification for using a sample of 250 is based on the recommendation of [47] who indicated that a sample of more than a hundred is a fair representation of a quantitative survey study. Another reason for choosing a sample of 250 is based on the convenience sampling technique which allows targeting of the relevance of respondents to provide relevant information relating to the issue under investigation.

### Instrument data collection procedures

Questionnaire became the sole data collection instrument. A primary reason is its ability to ensure maximum reliability and also provide responses that are more primary, factual and valid, especially from a larger sample [48]. It has been criticised for limitation in generating other “relevant” information that may not expressly be asked by the researchers. This drawback was checked in the current study by ensuring content validity.

All variables were measured on a five-point Likert scale ranging from 5 (strongly agree) to 1 (least agree) for research questions one and three. The scale for research question two ranges from 5 meaning very impactful to 1 meaning unimpactful.

This study employed the conceptualization of [49] who said that data collection is an “exact, systematic gathering of data significant to the research purpose”. Consequently, this study employed a quantitative survey approach to gathering quantitative responses from the respondents. An online google form was used to distribute the instruments. The initiative link was shared on the social media platform of the researchers where respondents were asked to participate voluntarily. The responses were automatically generated from the online platform and analyzed.

### Data processing and analysis

The responses generated were subjected to preliminary statistical procedures using IBM Statistical Package for Social Sciences (SPSS) version 21 where the data were coded, entered into the software and edited. The profile of the respondents was analyzed using descriptive statistics: frequencies and percentages. Objective one and two were analyzed using a descriptive-analytical framework using a histogram. Objective three was analyzed using a regression analytical approach to examine the relationship between service innovations (new service concepts, service processes and technologies) and customer loyalty.

### Ethical consideration

Both procedural ethics and ethics in practice were adhered to by the researchers. The necessary authorisations were received before data collection was conducted. Respondents’ consent was sought before their opinions were collected on the items of investigation. The introductory page of the data collection instrument expressly made provision for the respondent to decline or accept the continuation of the process. Additionally, respondents’ confidentiality and anonymity were assured before data collection began. The items of the study were carefully constructed to ensure that no psychological harm would be suffered by the respondents. Clearance was obtained from the College of Distance Education, University of Cape Coast.

### Validity and reliability

After the descriptive analysis of the profile, measurement items, and variables, the 206 final responses were tested for their validity and reliability to ensure that the variables under consideration give accurate meaning to what is expected to measure [50]. First, the reliability test was done using Cronbach alpha criteria [51]. This is consistent with the view of [52], that, validity and reliability are very important steps in research studies. Twenty measurement items were assessed based on the four variables used in this study. Table 4 therefore, presents the composite result of the validity and reliability tests. The result from Table 1 shows the reliability indicator above the satisfactory level greater than 70% as recommended by [51]. The alpha values for the four variables range from .840 to .935 confirming [51].

**Table 1:** Reliability and validity test result.

Measurement Item	Item Code	Factor Loadings	CA
**New Service Concept**			.935
“has creative service packages (voice, SMS and internet combinations)”	NSC1	.894	
“has flexible innovative service package options (client customization)”	NSC2	.843	
“is noticeably different in concept & design, compared to preceding services”	NSC3	.699	
“is a different service experience compared to preceding services”	NSC4	.894	
“is noticeably different in concept & design, compared to competing brands”	NSC5	.847	
**New Service Processes**			.877
“has online service options (procedures, support, usage history)”	NSP1	.750	
“has innovative automated service options”	NSP2	.857	
“provider offers quick and easy call centre support”	NSP5	.827	
**New Technology System**			.840
“has many innovative features (SMS to email, calling circles)”	NTS1	.712	
“provider offers the latest user equipment”	NTS2	.844	
“provider is always the first on the market with the latest technology”	NTS3	.837	
“is based on the latest technology applications”	NTS4	.756	
“provider shows its efforts for service quality improvement”.	NTS5	.712	
**Customer Loyalty**			.779
The “mobile call services (voice) are innovative so I will stay with them”	CL1	.661	
The “mobile internet services are innovative and I will be committed”	CL2	.772	
“Customer service is innovative and I will tell others about their services”	CL4	.818	

Source: Survey Data (2021)

Table 1 presents the validity and reliability of the test result.

The data dimension analysis also resulted in dropping one measurement item from the original seventeen questions. One out of 20 questions 16 loaded very well between .699 to .818. This category is above the 0.5 threshold and this confirms the validity of the study variables/data. The remaining 16 scales show all four variables were considered internally consistent and reliable for the study.

## Data analysis

This section shows the analysis of the study variables concerning the study’s objectives. First, a profile of respondents and a descriptive analysis of variables are presented. Table 2 shows the respondents’ profiles.

**Table 2:** Profile of respondents [n = 206].

Profile	Statement	Freq	(%)
Gender	Male	137	67
	Female	69	33
Age	18–25	78	38
	26–35	56	27
	36–45	31	15
	46–55	26	12
	55 and above	9	04
Highest education	JHS/SHS	10	05
	Certificate/Diploma	11	05
	Degree	101	51
	Post-graduate	51	48
	Others	29	14
Mobile Network	MTN	136	66
	Airtel/Tigo	27	13
	Vodafone	43	21

Source: Survey Data (2021)

The researchers also examined the descriptive statistics of the measurement questions which are used to collect the data. The reason for this analysis is to first, check the normality of the distribution of the data and describe how each of the specific questions is closer to the overall average of the measurement questions. Table 3 provides a descriptive analysis of 16 items of service innovations and customer loyalty.

**Table 3:** Descriptive statistics of measurement.

Measurements Items	Mean	Std. Dev.	Skewness
“has creative service packages (voice, SMS and internet combinations)”	3.23	1.20	-.560
“has flexible innovative service package options (client customization)”.	3.19	1.17	-.329
“is noticeably different in concept & design, compared to preceding services”.	3.18	1.05	-.335
“is a different service experience compared to preceding services”	3.23	1.20	-.560
“is noticeably different in concept & design, compared to competing brands”	3.19	1.17	-.339
“has online service options (procedures, support, usage history)”	3.92	1.24	-1.104
“has innovative automated service options”	4.00	1.07	-1.144
“provider offers quick and easy call centre support”	4.11	1.13	-1.371
“has many innovative features (SMS to email, calling circles)”	4.00	1.22	-1.271
“provider offers the latest user equipment”	4.07	.98	-1.006
“provider is always the first on the market with the latest technology”	4.10	1.01	-1.045
“is based on the latest technology applications”	3.98	1.20	-1.059
“provider shows its efforts for service quality improvement”	3.68	.99	-.687
“The mobile call services (voice) are innovative so I will stay with them”	3.48	1.20	-.418
“The mobile internet services are innovative and I will be committed”	3.46	1.20	-.306
“Customer service is innovative and I will tell others about their services”	3.26	1.10	-.206

Source: Survey Data (2021)

In Table 2, the item with the highest mean value was “provider is always the first on the market with the latest technology” *M* = 4.10, *SD* = 1.01. Whilst, the item “is noticeably different in concept & design, compared to preceding services” recorded the least mean value: *M =* 3.18, *SD =* 1.05.

**Fig 1:**
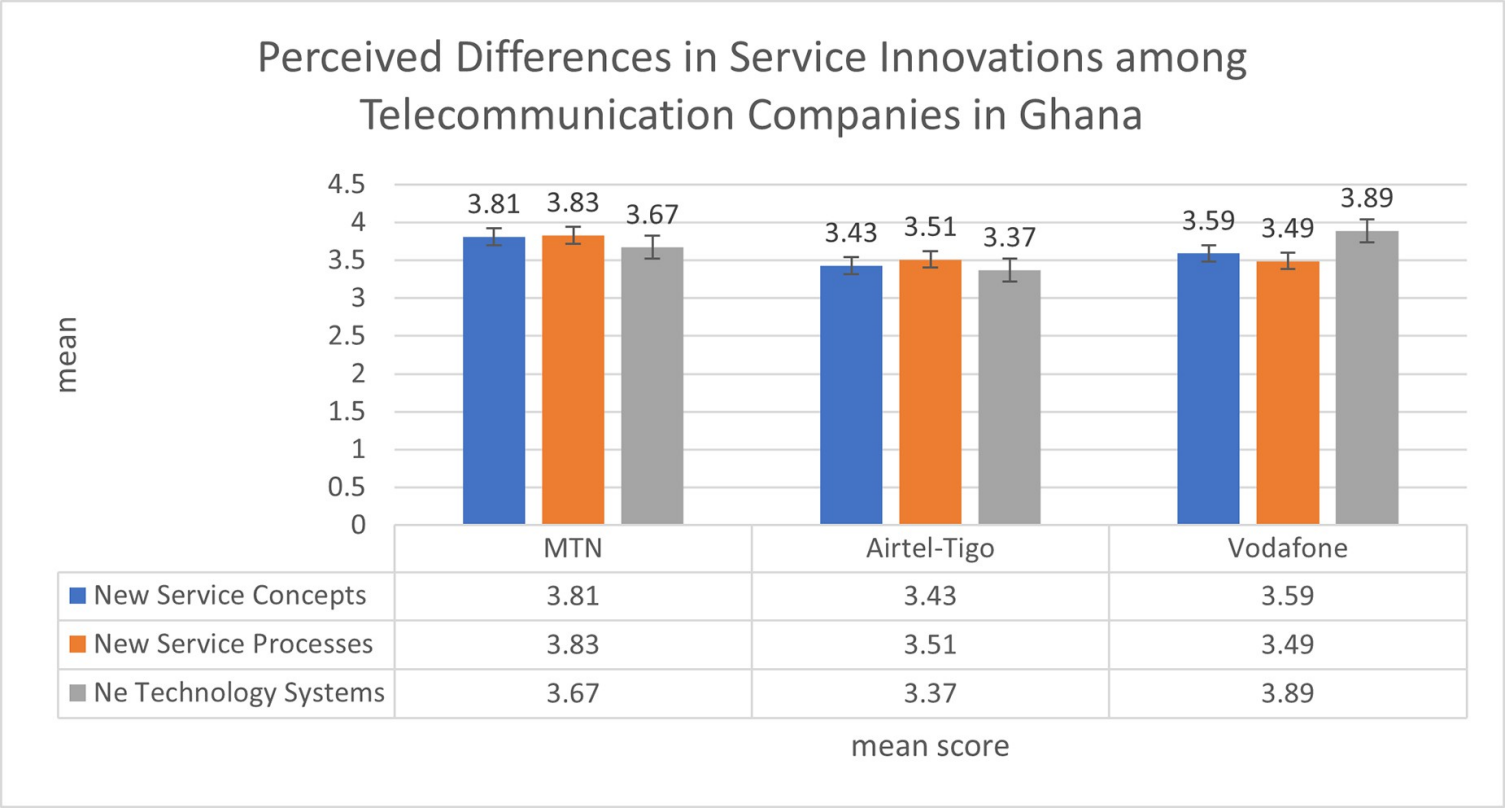
Perceived differences in service innovation among telecommunication companies in Ghana. Source: Field Survey (2021).

The first objective of this study assessed the perception of respondents about the differences in innovation practices of telecommunication companies in Ghana. Fig 1 shows the pictorial summary of customers’ perception of differences in innovation practices among MTN, Airtel-Tigo and Vodafone networks. The figure used mean values of the three main strands of innovations which were measured using a scale of 1 to 5.

The result in Fig 1 shows that respondents perceive MTN as better in terms of innovations in service concepts (Mean = 3.81) compared with Airtel-Tigo (Mean = 3.43) and Vodafone (Mean = 3.59). This means that MTN is better than Airtel-Tigo and Vodafone in terms of developing new and innovative services such as SME and internet packages, service options and service designs, and experiences compared with competing brands. On the other hand, Vodafone is more innovative in terms of developing new services for customers than Airtel-Tigo.

In terms of innovations in new processes, the study found that MTN is more innovative (Mean = 3.83) compared to Airtel-Tigo (Mean = 3.51) and Vodafone (Mean = 3.49). This means that MTN is more innovative in terms of developing new automated service options, and innovative interactive media, and provides innovative mobile shops and quick call centre support for subscribers. In this regard, Airtel-Tigo is more innovative than Vodafone.

Thirdly, the result shows that in terms of the innovative technologies in the delivery of mobile telecommunication services, Vodafone is more innovative in technologies (Mean = 3.89) compared to MTN (Mean = 3.67) and Airtel-Tigo (3.37) networks. This means that Vodafone is more innovative in terms of developing innovative technology applications, and service features to deliver service and serve customers. In technology innovation, MTN is more innovative than compared to Airtel Tigo.

In summary, the descriptive statistics on the consumer perception of service innovations of three active mobile networks in Ghana show that MTN is more innovative in terms of innovativeness in new service concepts and service processes. In terms of new service technologies, Vodafone is more innovative than MTN and Airtel-Tigo.

The next section assesses the perception of the service innovation practices and whether their implementation impacts the users of the mobile networks.

**Fig 2:**
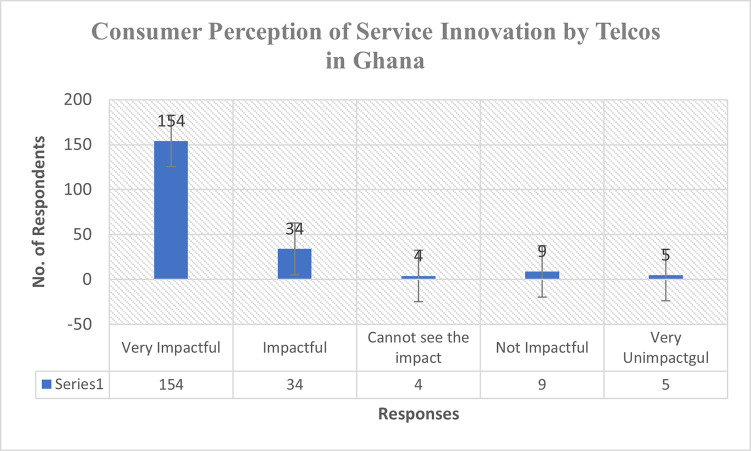
Customer perception of service innovations by Telcos in Ghana. Source: Field Survey (2021).

The second objective of this study assessed the perception of respondents about service innovation practices in the telecommunication industry. Fig 2 shows the pictorial summary of customers’ perceptions the service’s innovative practices.

The result as in Fig 2 showed that the majority of the mobile subscribers perceive that service innovations by the telecommunication companies in Ghana have an impact.

From the data, a total of 154(74.7%) respondents perceive service innovation practices by the three telecommunication companies are “very impactful”. This is followed by a total of 34(16.5%) who perceive service innovation practices by the three companies as “impactful”. The study results further show that 9 (4.3%) and 5(2.4) of the respondents perceive that service innovation practices were “unimpactful” and “very unimpactful” respectively. The effect of this result relative to study objective two is that 188(91.2%) of the respondents perceive that service innovation practices by telecommunication companies in Ghana are impactful. Fig 2 presents the pictorial evidence of the result.

Objective three of the research investigated the influence of service innovation practices and customer loyalty of the three telecommunication companies in Ghana. It is recalled from the validity and reliability data analysis that, fifteen measurement items were confirmed and transformed into four variables. Three of the variables namely new service processes (NSP), new service concept (NSC) and new technology system (NTS) are independent variables. The dependent variable is customer loyalty (CL). Tables 4–6 present the result from the regression analysis of research objective three. First, Table 4 shows the effect of a change in customer loyalty due to a unit change in the overall service innovation. Skewness was assessed which shows the normality, thus confirming suitability for regression. Also, the study leverages the central limit theorem based on the large sample size to conclude that the data is normally distributed.

**Table 4:** Model summary of service innovation practices and customer loyalty.

Model	R	R Square	Adjusted R Square	Std. Error of the Estimate
1	.992^a^	.983	.983	.12552

a. Predictors: (Constant), New technology system, New service concept, new service process

Source: Field Survey (2021)

**Table 5:** ANOVA of service innovation practices and customer loyalty.

Model		Sum of Squares	df	Mean Square	F	Sig.
1	Regression	185.131	3	61.710	3916.487	.000^b^
	Residual	3.183	202	.016		
	Total	188.313	205			

a. Dependent Variable: Customer loyalty

b. Predictors: (Constant), New technology system, New service concept, new service process

Source: Survey Data (2021)

**Table 6:** Hierarchical result of service innovation and customer loyalty.

Model		Unstandardized Coefficients		Standardized Coefficients	t	Sig.
		B	Std. Error	Beta		
1	(Constant)	-.212	.043		-4.877	.000
	New service concept	.026	.011	.025	2.310	.022
	new service process	.249	.011	.269	23.482	.000
	New technology system	.778	.010	.815	74.811	.000

a. Dependent Variable: Customer loyalty

Source: Survey Data (2021)

The result in Table 4 shows a 98.3 percent variation between overall service innovation practices and customer loyalty as explained by the adjusted R-Squared. This statistic means that a unit change in service innovation by telecommunication has the likelihood to change customer loyalty by 98.3 percent. This finding is important because it shows that service innovations are important and can influence customer loyalty. Table 5 shows the level of significance between service innovation practices and customer loyalty.

Table 5 shows the Analysis of the Variance between service innovation practices and customer loyalty. The statistics in Table 6 show the level of significance between employee orientation and productivity. Analysis of the result shows a significant positive effect of service innovation practices on customer loyalty (*F* = 3916, *p*< 0.05). The result implies that in a cumulated sense, service innovation leads to customer loyalty. These findings are important because it confirms that service innovation practices of telecommunication companies significantly impact customer loyalty. The next section of the analysis presents the extent to which each of the service innovation practices impact customer loyalty. The result is presented in Table 6.

Table 6 present the hierarchical coefficient result which shows how each of the service innovation orientations influences customer loyalty. The table also shows the magnitude of the significance between each of the three strands of innovation and customer loyalty. The research proposed that innovations in new service concepts (H_1_), new service processes (H_2_), and new technology systems (H_3_) significantly influence customer loyalty. The results confirm three hypotheses (H_1_; H_2_; H_3_). Confirming the hypotheses, the result means that innovations in new service concepts (H_1_: β = .295; p-value = *** < 0.*05*), new service processes (H_2_: β = .325; p-value = *** < .*05*), and new technology systems (H_3_: β = .159; *p-value =* .*014 <* .*05)* significantly and positively influence the loyalty of mobile subscribers. This means that introduction of innovative new service processes, new technology systems and new service concepts significantly influence the loyalty of subscribers of the three mobile networks.

The result also shows that among the three service innovation practices new technologies and innovations from the telecommunication companies (H_3_: β = .815) has the strongest influence on customer loyalty. The statistics mean that when telecommunication companies change technological innovation (improve or reduce) by one unit, subscribers’ loyalty could also change (improve or reduce) by 81.5 percent. This is followed by new service innovations (H_1_: β = .269) and new service concepts (H_3_: β = .025).

## Discussion

This section of this research chapter provides a discussion of the finding. The analyses relate the findings to the empirical literature. Again, the discussion is presented based on the three main objectives of the research.

This current research revealed that among the three mobile networks that were used for this study, they all undertake some service innovations. These innovations are principally found in the innovation spheres of introducing new service concepts, new service processes and new technology systems. Among these three strands of innovations, MTN is more innovative in developing new service concepts and new processes. The researchers perceive that the reason may be due to the high patronage of MTN and the public perception that MTN is the leader in the telecommunication industry. The study also found that Vodafone is more innovative in introducing new systems to deliver a service delivery framework. This result corroborates previous evidence by [33, 35] who posited that innovation practices differ from firm to firm and from industry to industry.

The second objective assessed the perception of service innovation practices by three telecommunication companies that were used in this study. The result shows that the majority of respondents perceive service innovation by the three telecommunication companies as impactful. The researchers believe that the responses resonate with the high-level acceptability of some of the innovations that the telecommunication operators have introduced such as Mobile money services. This is similar to previous evidence in the literature that found evidence that innovation emerging from complete or partial changes and modifications in processes, output and structure could lead to impactful outcomes [23]. The findings also corroborate the work of [37, 39] who posited that different innovations may produce a different level of impact depending on the value of such innovations to the consumers and how the innovations are delivered to the final consumer.

The third objective investigated the influence of service innovation practices on customer loyalty. The study found that a change in innovation practices could influence loyalty by 98.3 percent. Again, the study confirmed that service innovation practices influence the loyalty of mobile subscribers. Analysis of the data shows a significant positive effect of service innovation practices on customer loyalty. The result implies that innovation in the service industry drives customer loyalty, which lends empirical credence to [53] who found that effective and innovative service delivery could entice consumers to be loyal.

The study also found that new technologies and innovations in the service delivery framework have the strongest influence on customer loyalty followed by new service innovations and new service concepts.

First, in terms of new service concepts, the result suggests that developing new service packages such as SME and internet services, and providing innovative service options, designs, and experiences which are better than competing brands could influence customer loyalty. Second, in terms of new service processes, the result suggests that developing innovative automated service delivery options, creating innovative interactive media for customers, innovative mobile shops and quick call centre support motivate mobile subscribers to remain loyal to the brand. Third, in terms of new technologies, the result suggests that developing innovative technology applications, designs and service features to deliver and serve customers entices them to remain loyal to a mobile service provider.

The result confirms previous studies such as [9, 54] who found that service innovations lead to a better service delivery system that drives customer loyalty. The findings are also similar to [23] who found that service innovations drive customer loyalty. Thus, the findings from this study have confirmed that constant innovations that lead to new products, idea and service features could lead to customer repurchase intention which further collaborates with evidence by [42, 43].

## Conclusion

The following conclusions have been drawn based on the objectives and findings from this investigation. These fill the research gaps identified earlier. First, the study concludes that MTN, Vodafone and Airtel Tigo telecommunication brands in Ghana undertake service innovations. These innovation strands include developing new service concepts, new service process systems and new service technologies aimed at driving customer loyalty. However, MTN is more innovative than Vodafone and Airtel-Tigo in terms of developing new service concepts and processes while Vodafone is more innovative than MTN and Airtel-Tigo in terms of developing innovative technologies for customers.

Mobile subscribers of MTN, Vodafone and Airtel Tigo perceive that the service innovation practices by the three companies are very impactful and they significantly influence their loyalty. Consequently, a change in service innovations could predict a 98.3 percent change in the loyalty of mobile subscribers. Innovative service concepts, innovative processes and technologies all influence loyalty of mobile subscribers. However, technological innovations in the service delivery processes have the most influence on subscribers’ loyalty followed by innovative service processes and service concepts. The high predictive power of service innovation practices on loyalty correlates with the foundational premise of destructive innovation theory. The theory makes meaning in the telecommunication service industry.

## Recommendations

Several managerial implications can be drawn from the findings. These practical implications are relevant to managing the service innovation strands to achieve maximum customer loyalty which will also influence the performance of the company. From the research findings, it came to fore that service innovations can improve customer loyalty when consumers perceive their innovations as impactful. This means that telecommunication companies must develop innovative service technologies, products and processes to deliver superior services to customers.

By implication, the management of MTN, Vodafone and Airtel-Tigo in collaboration with their R&D and Marketing departments must invest financial and cognitive resources to develop innovative technologies, processes and services to address the service convenience, efficiency and effectiveness needs of customers. The investment should focus on market and consumer research, and also increase their interaction with customers. The outcome of such investment should produce innovation that will put the brand on top of the consumers’ minds.

Furthermore, the findings also suggested differences in the impact of innovations on customer loyalty. Therefore, investment in the new service process, new service concept and new technological systems should be properly focused and planned to derive the maximum return. Given this, telecommunication companies surveyed in this study should consider the multi-dimensional impact of various innovations that customers will consider more impactful. Findings from this study suggested that innovations in service technologies such as developing innovative applications, designs and service features and equipment can provide more significant benefits in terms of driving customer loyalty.

This study has made some modest contributions to the literature on innovations in the telecommunication industry. Thus, this study has contributed to closing the gap in the limited literature on innovation which is mostly concentrated in the manufacturing sector. The study has synthesized the literature in the field of innovation to provide empirical knowledge on the impact of service innovation on customer loyalty in the service sector. This empirical knowledge provides researchers, policymakers and practitioners with empirical grounds that service innovation boosts customer loyalty.

## Future research directions

This research has some limitations which provide an avenue for future research direction. First, the investigation focused on the three mobile telecommunication companies in Ghana. Future investigations may consider other service providers in the service industry such as the insurance and banking institutions and make comparisons to provide a broader perspective on the issue. Based on the design that was employed, the study was unable to explore other strands of service innovation that could influence customer loyalty. An exploratory study is recommended to explore other possible strands of innovation. Additionally, no mediation or moderating effect was tested. Future studies may consider such an analysis.
